# A conceptual model of physician-patient relationships: a qualitative study

**Published:** 2016-11-08

**Authors:** Mohammad Reza Razzaghi, Leila Afshar

**Affiliations:** 1 Professor, Laser Application in Medical Sciences Research Center, Shahid Beheshti University of Medical Sciences, Tehran, Iran;; 2 Assistant Professor, Department of Medical Ethics, Shahid Beheshti University of Medical Sciences, Tehran, Iran.

**Keywords:** *Physician-patient relationship*, *Healing relationship*, *Qualitative research*, *Islam*

## Abstract

In any clinical encounter, an effective physician-patient relationship is necessary for achieving the desired outcome. This outcome is successful treatment, and therefore, the relationship should be a healing one. In addition, in the Islamic view, the physician is a manifestation of God’s healing attribute, which is usually undermined in everyday therapeutic communications. Yet there are few empirical data about this experience and how it occurs in the clinical context. This study was conducted to develop a model of physician-patient relationship, with the healing process at its core. Our goal was to explain the nature and characteristics of this encounter. In Islamic teachings, healing is defined as “cure” when possible and if not, reducing pain and suffering and ultimately finding a meaning in the illness experience.

This study was a qualitative inquiry. Data were collected through 17 open-ended, semi-structured interviews with physicians who had an effective relationship with their patients. The participants’ experiences and their perception regarding the relationship were subjected to grounded theory content analysis. For establishing the trustworthiness of the data collection and analysis we used triangulation, peer review, and member checking.

The findings showed that the components of the patient-physician healing relationship could be categorized in the four key processes of valuing the patient as a person, effective management of power imbalance, commitment, and the physician’s competence and character. This leads to forming the three necessary relational elements of trust, peace and hope, and being acknowledged. Their importance has been better demonstrated in a relationship which incorporates the spiritual aspects of patient care and also physician’s satisfaction.

The physician-patient relationship has a central role in patient outcome. This relationship has an understandable structure and its components may have an effective impact on promoting the patient’s experience of the health system.

## Introduction

The physician-patient relationship is a clinical encounter and its effective outcome has a direct impact on the quality of care (1) and achievement of a successful treatment. Hence, a healing relation must be formed between a physician and his/her patient. However, today, the physician-patient relationship undermined and the medical professional is considered as the technician and the patient is considered as the machine (1). Nevertheless, the establishment of an effective communication with the patient requires an understanding of the fact that the patient is not just a set of symptoms of a damaged organ, but a human with dignity and his or her own concerns and aspirations, who is in search of help and healing. The importance of such an effective therapeutic relation should not be ignored. Moreover, in most cases, accurate diagnosis and effective treatment of the patient are directly dependent on the quality of the mentioned relationship. The development of such a relationship would allow the patient to share his/her most personal and private information with the physician in a safe and intimate environment.

The scientific impact of the common philosophy of medicine which underestimates the patient’s body to a set of organs cannot be denied since such an approach has led to many developments in the field of medicine (2). Although dissenting voices are heard in modern medicine, there has been clear evidence of the importance of this relationship in the ancient medical history of Iran, especially in the Islamic period (3). This strong background gives us the opportunity to explain and design a model of physician-patient relationship.

Human relationships have a crucial status and people’s rights, good conduct, and trusteeship have been emphasized in Islam. Islam considers the physician-patient relationship as a value-based and sacred one, because of knowing the physician to be a manifestation of God's healing attribute. The goal of the physician-patient relationship, as any other relationship, is maturity, integrity, and excellence (4, 5).

During the past decades, there has been a struggle over the physician-patient relationship and its impact on the medical decision-making process, and so, on the therapeutic outcome. This struggle is often defined as a conflict between patient’s autonomy and his/her health in other words, the values of the patient and the values of the health care providers. Based on this conflict, four models of physician-patient relationship have been proposed (6). However, based on the type of relationship, its purpose, effectiveness of the physician’s role, and the existing expectations, each relationship between a physician and his/her patient is unique (7). This relationship is also influenced by cultural elements (7). Yet, we know little empirically about this experience and how it occurs in the clinician-patient relationship. For this reason, it is necessary to redefine and design the specific relational model of every society based on its own components.

The core of the medical practice is communication and the purpose of this communication is patient’s healing and satisfaction and physician’s integrity. On this basis, all elements of the relationship should be formed in line with the ultimate goal of medicine, i.e. the best and most righteous possible action for each patient. This means that the nature and characteristics of the physician-patient relationship can be drawn through identification of its fundamental concepts and based on these elements a conceptual model could be proposed. The impacts of the cultural and value system on human relationships provide the possibility of designing a model based on an Iranian-Islamic pattern for therapeutic relationship.

In this study, healing was defined as cure if possible, and when cure is not achievable, trying to reduce the patient’s suffering and or finding a meaning in the patient’s experience. This study was conducted to explore the positive effect of the physician-patient relationships. Our focus on physician-patient relationships is because of its potentiality to improve the physician’s professional conduct and the patient’s satisfaction.

## Method

The aim of this study was to explain and propose a physician-patient relationship model and its components. The focus of the study was healing relationship due to its potential capacity to enhance the patient’s satisfaction and physician’s integrity. Therefore, the qualitative paradigm was chosen and utilized. Data were collected through 17 open-ended semi-structured interviews with physicians who had effective relationships with their patients. The physician’s perception regarding the relationship was subjected to grounded theory content analysis. 

The participants were selected from different medical specialties and the sampling process continued until data saturation. The physicians were selected based on the quality of their relationship with the patients who were the exemplars in this issue.

Data collection was performed through semi-structured interviews. For this purpose, an interview guideline was prepared with a number of general open-ended questions to help the researcher. The objective of the research and its methodology were explained to each participant in a separate meeting before the interview and their oral informed consents were obtained. The place and time of the interviews were determined by the participants and the durations of the interviews varied between 60 and 90 minutes based on participants’ willingness.

After each interview, the researcher documented all recorded files and field notes. All statements and expressions of the participants were completely rewritten verbatim in Microsoft Word and coding was done on three levels. Through this method, the initial codes, sub-categories, and themes were extracted from the data.

It is worth noting that the first step of analysis of initial codes was extracted from a thousand semantic unites. In later stages, the codes were gradually reduced by removing the identical and overlapping codes and they were gradually categorized into the classes and sub-classes explained in the following sections (8, 9). Data-source triangulation was performed by collecting data from physicians who practice in different fields. For establishing the trustworthiness of the data collection and analysis we used triangulation, peer review, and member checking. An expert colleague in qualitative methods examined the interview transcripts, coding sheets, and the synopsis of the findings. In this peer review process the analysis was identified complete and logical, based on the interview sheets, the study findings were accurate. For the member checking 2 of the participants revised the data and affirmed that the findings were consistent with their experiences.

***Results***

In this study, according to the data transcription, saturation was achieved after 17 interviews.

Participants were specialists in fields that naturally dealt with chronic or life-threatening diseases and had effective relationships with their patients over time. 

**The physician-patient relationship in the healing process**

Based on the findings of this study, which are provided entirely in table 1, the physician-patient relationship model consists of four components. These components conduce to three relational outcomes, all of which come under a common theme.

**Table 1:** Physician-Patient relationship components

**Category**	**Sub-category**	**Definition**
Valuing the patient	Non-judgmental behavior	Accepting the patient as a person
	Communication	Effective communication with patients based on their individual character
	Effective presence	Effective listening, appreciating the patient’s experience of illness and empathy
The balance of power	Shared decision-making	Engaging patients in diagnostic and therapeutic decision-making
	Educating the patient	Explaining medical terminology in an understandable language for the patient and educating them to follow the care tasks
	Guidance	Using authority to encourage the patient to follow medical advice
Commitment	The continuity of the relationship	Constant communication with patients over time
	Presence	Being present in critical situations
	Fidelity	Being present even if cure is not possible
Physician’s character and competencies	Self-confidence	Physician’s confidence in the the diagnostic and therapeutic plan
	Managing the emotions	The ability to be aware of his/her emotions and control them
	Mindfulness	The ability to consider the internal and external forcible factors
	Scientific knowledge	The physician’s expert knowledge

**Components of the physician-patient relationship model**

*Valuing the patient*

The first component of the model is the amount of value which the physician considers for his/her patient. This category includes three sub-categories of non-judgmental behavior, connecting with the patient, and effective presence. The valuing process begins with a non-judgmental stance and respecting the patient’s dignity. This includes all patients from different cultural and social classes and with different diseases. It was stated by one of the participants: [*I have the same attitude toward all my patients with different cultural levels ..... My colleagues have often told me that you cannot work in this cultural community, but I want to do my valuable work and it does not matter for me where I am working.*] Another interviewee stated: [*As a physician, I cannot transfer my message correctly unless I have come to understand and believe that I am responsible for the health of people who are sitting in front of me, and I must respect it*.] Another participant said: [*We have to respect patients whether they are children or adults.*]

Participants also said that building an effective communication requires the consideration of the characteristics of the individual patient and acknowledgment of common humanistic experiences. In this regard, one participant said: [*Children sometimes say we do not want to see another physician until you come back; I have nothing to give them it is just an emotional relationship.*] Another participant stated: [*I always try to build an empathetic relationship with my patients and their family, it helps develop trust*.]. This type of relationship results in the consideration of the patient as a person and not just a diseased body. In this regard, one physician indicated: [*I must be both physically and psychologically helpful for my patients.*] In fact, this type of treatment results in an integrated picture of the patient as body, mind, and spirit.

The third sub-category of valuation is effective presence at the bedside of patients or full attention to them. In this regard, one participant mentioned: [*I propose complete care for my patients with full attention; this will prevent many problems.*]

The full and active presence of the physician in the clinical encounter will result in a good relationship and trust. As one participant stated: [*When you speak with the parents, calming the mother, this simple activity changes everything ..... Sometimes just active listening is enough…. to create an empathetic relationship*.]

Furthermore, understanding the patient’s experience of illness and suffering requires the physician’s full presence. This will enable the physician to communicate with patients in an empathetic manner.

[*I feel a sense of peace*" another participant said: "*If patients feel that you are tired or you are not fully present, they will not trust you and the treatment will not be effective*.]

*The balance of power*

The participants of this study reported that they had often tried to empower the patients or their family. Sometimes, this has been done by engaging the patients in therapeutic decision-making. As one said: [*Explaining the issues to the patients or their family is very effective and often results in their following the treatment*.]

Another way to prevail over the power imbalance is educating the patients. This begins with the explanation and translation of medical terminology into a comprehensible language for the patient and educating them to follow the care tasks. One participant remarked: [*I tell patients that they have to connect with me every week, even after finishing the drugs. The patient’s health status will improve with only simple recommendations.* *Because it is my verbal communication that will assure the patients; and in this method I will educate my patients*.] Another participant said: [*I have to talk with each patient in their own language. The doctor should have the skills to convey his message.*]

Sometimes it is necessary for physicians to use their power and authority to encourage the patients who do not accept responsibility for their treatment. For example, one participant mentioned: [*I say to my patients that if you think you can trust me, listen to me and obey my recommendations, and then, we will move ahead together*.] Physicians who participated in this study mentioned that they intuitively understand with which patients and at what time it is necessary to use their power and authority. This depends on the patient’s needs and the type of physician-patient relationship.

*Commitment*

The common feature of physicians participating in this study was working in one place for years. This leads to constant communication with the patients. This form of communication between a physician and patients, in turn, creates an intimacy that it is called a sense of family relationship. In this regard, one participant said: [*I always tell to my students that you should think the patient is your sister*.]. Another participant asserted: [*I think doctors need to have a sense of a close relationship with the patient.*]

The experience of a long-term relationship between the physician and his/her patient, especially in health crisis events, leads to the formation of a common history, and thus, a strong and rich therapeutic relationship between them.

Doctors show their commitment to their patients through care activities. Some of these activities, in our current medical context, are extraordinary, like home visiting or phone calls. Others fit in the context of routine daily actions; however, in their opinion these activities are signs of respect for the patient.

For example, one participant noted: [*When a patient is seriously ill, I may call him/her ten times a day to ensure his/her wellbeing*.]

Another participant said: [*I visited a patient who could not move at home, then, I gave him my phone number, and based on my experiences, step by step I told him what to do and this led him to trust me, and his outcome was much better than the expectations*.]

Another participant said: [*One of the things I do is that each day after seeing the last patient in my office, I call all patients who have called me. This results in them trusting me*.]

These caring and supporting activities reflect the commitment of physicians to their patients, which, in addition to medication and medical technologies, could influence the outcome of the healing process.

*Physician’s character and competencies*

If the physician is confident of his/her diagnosis and treatment, this confidence will effectively transfer to the patient. This self-confidence is an important component of the healing relationship. The physician’s self-confidence will led to a sense of confidence in the patient.

The physician’s ability to manage and control his/her emotions needs some fundamental skills such as understanding their own emotions and having appropriate emotional reactions to patients in order to keep calm. In this regard, one of the participants stated: [*Management does not mean that a person is the manager of an office; he/she should manage him/herself*.]

Mindfulness is another important feature that is defined as continuous awareness of what is happening at different levels. It means to be able to recognize if the patient’s words originate from his/her deepest emotions of fear, anxiety, regret, or guilt.

Finally, expert knowledge is important in building an effective communication. The patient must be able to trust the physician’s knowledge and skills, and at the same time must understand the physician’s limitations and the necessity of consulting with other medical practitioners. In this regard, one participant said: [*Even if the patient dies, the patient's family knows that I have done all I had to; I have consulted with everyone regarding what was necessary*.]

***Relational outcomes***

If the mentioned components are applied appropriately, trust and peace and hope are achieved, and the patient is acknowledged, which are called relational outcomes and are necessary for the healing process.

*Trust*

 Trust will form as a result of the process of valuation and commitment over time. One of the participants stated: [*The patients must find their trusted physician; otherwise, they will not have faith in the medications and treatment*.]

The patients’ trust in their physicians does not mean that medical errors will not occur, but this trust indicates that the physician has done all that is within his/her power and at the same time acknowledges his/her limitations. One subject remarked: [*The patient understands that I have done all I can, even if the desired outcome was not achieved.*]

*Peace and hope*

A therapeutic relationship can bring peace to the patients and their families. This does not always mean that cure is possible, but sometimes it means that there is hope of a few more good days, and provides opportunity for gratitude and forgiveness.

In difficult situations, physicians act differently. In an effective communication and healing relationship, the patient feels the physician’s empathy and sympathy and understands it. They do not seek false hope; however, the patient will have a pleasant experience unless there was no effective treatment.

In this regard, one participant said: [*If we could explain the nature of the disease to the patient’s family and if we could explain death, then, they would be ready to go. They could understand where they were going and what was going on*.]

*Feeling the physician’s attention*

Another outcome of effective communication is the patients’ sense of being acknowledged. This feeling results from valuing the patient and is based on the physician’s commitment. In such a situation, the physician recognizes the patient as a person and remembers him/her. In this regard, a physician stated: [*The better the physician and patient know each other, the more desirable the outcomes achieved* will be.]

***Theme***

As noted above, to understand the foundation of an effective healing relationship, we should consider the cultural context. Accordingly, in the Iranian society, mentioning the Islamic concepts of human relationship can improve the quantity of health relationship and can enrich the components of the physician-patient relationship. In this context, a basic element and a prerequisite to respecting human dignity is viewing every human being as a creation of God. In this regard, one participant said: [*When you cannot do anything for your patient, it is time for spiritual care ..... This means that it is your intent that shapes the relationship*]. Another participant stated: [*A small part of the therapeutic relationship is physical; the more profound a physician’s perception of his work is, the deeper his relation with his patients will be and it will be a part of his life*.] Another participant said: [*Spiritual and emotional pleasures are constant and are associated with our religious beliefs*.] Another physician said: [*I believe that this work is God’s desire, it is God’s kindness that the patients’ need our knowledge and skills*.]

## Discussion

The findings of this study showed that physicians, who had experience of effective communication with their patients, understood the therapeutic relationship and healing process in almost same way. In their view, healing means cure when it is possible, and reducing patient’s suffering and finding a meaning beyond the illness experience when cure is not possible.

The focal center of this healing relationship is neither the physician nor the patient, but is their therapeutic relationship or “the between” (10) and is based on the common underlying beliefs, trust, and hope (11). This therapeutic relationship consists of understandable components which are valuing the patient, commitment to the patient, managing the power imbalance, and physician’s character and competencies. These components will form the three relational elements of trust, peace and hope, and being acknowledged.

The present study tried to propose a model for therapeutic relationship based on the experiences of the research participants.

In order to determine the effect of this model in the current situation of medical practice, we can evaluate it at three levels. First, the therapeutic physician-patient relationship could enhance the patient’s quality of life. Mutual trust, peace, and being acknowledged as the results of this relationship are equally important for both patients and physicians.

Second, some aspects of this relationship are related to the nature and severity of the disease and the type of required treatments (12, 13); however the patient’s and physician’s system of belief also play an important role in this regard (14).

Third, the healing relationship affects both parties (patients and physicians). A number of physicians who participated in this study had experienced working in difficult situations (such as the battlefield or severely deprived areas) and they were satisfied with memorizing them. This experience is opposed to the cases that are reported from the physicians’ current behaviors (15). This indicates that such an enriched experience and relationship left its positive impact on their life and work.

Finally, it can be said that an effective and healing relationship is based on a system of belief that acknowledges the value of the human being and his/her inherent dignity, and accordingly considers clinical medicine as a favor which gives meaning to the physician’s life. It could be proposed that the solution to some health system problems could be found in redefining the physician-patient healing relationship.

## Conclusion

It can be concluded that the structure of the physician-patient healing relationships is comprehensible and may lead to valuable patient-centered outcomes. Moreover, this discernible and understandable structure has important impacts on treatment. Furthermore, this conceptual model can be generalized to other therapeutic relations in the health system.

Evidently, this study had some limitations. First, the participants were chosen selectively and are not considered significant indicators of the population of physicians. Nevertheless, it can be explained that the intention of this study was to provide a preferred model of doctor-patient communication, and for this purpose, it was necessary to choose a targeted selection of participants. Accordingly, the proposed model of the study does not explain the current situation, but if experimental studies confirm its effectiveness, it could be a standard to achieve.
